# On-Demand Centralized Resource Allocation for IoT Applications: AI-Enabled Benchmark

**DOI:** 10.3390/s24030980

**Published:** 2024-02-02

**Authors:** Ran Zhang, Lei Liu, Mianxiong Dong, Kaoru Ota

**Affiliations:** 1 School of Software, Shandong University, Jinan 250101, China; zhran@mail.sdu.edu.cn; 2 Department of Information and Electronic Engineering, Muroran Institute of Technology, Muroran 050-8585, Japan; mx.dong@csse.muroran-it.ac.jp (M.D.); ota@csse.muroran-it.ac.jp (K.O.)

**Keywords:** IoT, resource allocation, performance prediction, benchmark, artificial intelligence

## Abstract

The development of emerging information technologies, such as the Internet of Things (IoT), edge computing, and blockchain, has triggered a significant increase in IoT application services and data volume. Ensuring satisfactory service quality for diverse IoT application services based on limited network resources has become an urgent issue. Generalized processor sharing (GPS), functioning as a central resource scheduling mechanism guiding differentiated services, stands as a key technology for implementing on-demand resource allocation. The performance prediction of GPS is a crucial step that aims to capture the actual allocated resources using various queue metrics. Some methods (mainly analytical methods) have attempted to establish upper and lower bounds or approximate solutions. Recently, artificial intelligence (AI) methods, such as deep learning, have been designed to assess performance under self-similar traffic. However, the proposed methods in the literature have been developed for specific traffic scenarios with predefined constraints, thus limiting their real-world applicability. Furthermore, the absence of a benchmark in the literature leads to an unfair performance prediction comparison. To address the drawbacks in the literature, an AI-enabled performance benchmark with comprehensive traffic-oriented experiments showcasing the performance of existing methods is presented. Specifically, three types of methods are employed: traditional approximate analytical methods, traditional machine learning-based methods, and deep learning-based methods. Following that, various traffic flows with different settings are collected, and intricate experimental analyses at both the feature and method levels under different traffic conditions are conducted. Finally, insights from the experimental analysis that may be beneficial for the future performance prediction of GPS are derived.

## 1. Introduction

The rapid growth of Internet of Things (IoT) technology has brought an increase in IoT application services and data volume. Specifically, a wide array of physical devices connected to the IoT network has resulted in an exponential increase in the number of devices generating data. The diverse applications in areas such as healthcare, agriculture, and transportation have yielded a wide range of IoT data types and use cases [1,2]. Different IoT application services have different requirements for the quality of service (QoS) [3]. Ensuring satisfactory service quality for diverse IoT application services is a critical issue, especially considering limited network resources. Specifically, the diverse nature of IoT applications makes it challenging to adopt a one-size-fits-all approach to ensuring service quality. For example, many IoT applications, such as autonomous vehicles, demand real-time data processing and low-latency communication. Delays or disruptions in service can have severe consequences in these mission-critical scenarios. In contrast, mobile phones are relatively delay-tolerant and bandwidth-tolerant, as small packets are delivered [4]. In the context of limited resources, allocating too many resources to phones and too few resources to autonomous vehicles will result in wasted resources for mobile phones and dangerous consequences for autonomous driving applications. Therefore, network resource allocation in terms of different requirements for the QoS has always been a hot topic. To allocate network resources among different application services in terms of the corresponding QoS requirements, fair resource scheduling has drawn broad attention [5,6,7,8]. Fair means providing the QoS on demand.

Generalized processor sharing (GPS) scheduling, as the fairest scheduling mechanism with the byte as the minimum scheduling unit, has been widely utilized as the fairness guidance for central resource scheduling. Specifically, GPS scheduling functions as a central resource scheduling mechanism, as it enables different application services to share the network resources (such as the service capability of a server or an entire cloud service center). GPS scheduling allocates the network resource to different application services based on the weight assigned to each application service through scheduling the application requests. In addition, by comparing the QoS for differentiated services achieved in the resource allocation system and the fair QoS obtained under the GPS scheduling mechanism, GPS scheduling guides differentiated services for on-demand resource allocation. To be suitable for a variety of application services, multi-queue GPS has been mainly studied. As long as the QoS obtained by each application service is not worse than that obtained using the GPS scheduling mechanism, the resource allocation is regarded as fair and can provide differentiated services on demand.

The performance prediction of each queue in multi-queue GPS corresponding to each type of application service is of significance for fair scheduling. First, the performance prediction of the GPS enables the provision of QoS guarantees to different IoT applications. Knowing how resources will be allocated helps in setting appropriate weight assignments to ensure fairness and meet the QoS for different application services. Second, the performance prediction of GPS allows for dynamic adjustments of weights for IoT application services based on different QoS demands, which helps in adapting to changing network conditions and traffic patterns. Third, network administrators can optimize the configuration of the GPS and other scheduling algorithms to achieve better overall network performance through performance prediction. In addition, it is also crucial to capture actual allocated resources using various queue metrics. Specifically, different queue metrics provide information about different aspects of the network, such as the total queue length for all application services and the queue length for each application service. Specifically, the total queue length reflects the overall congestion situation of the scheduling system, which can quickly locate the congested server or cloud service center, but it cannot determine the specific data source of congestion. The queue length of each application reflects the congestion of each application requesting the server. By monitoring the queue length of each application, the resource allocation weight of the application can be adjusted appropriately, or the flow of the application can be limited to relieve congestion.

Many researchers have focused on the performance prediction of the multi-queue GPS scheduling mechanism. Existing methods mainly predict the performance metrics using analytical methods and deep learning-based methods. For example, in [9,10], the authors employed large deviation principles-based (LDPs) analytical methods to derive the total queue length distribution for the overall GPS scheduling. In [11], the authors used empty buffer approximate (EBA)-based analytical methods to derive the queue length distribution of each application service. In addition, to obtain accurate performance metrics, Zhang et al. [12] tried to predict the performance of the GPS scheduling mechanism by employing a deep learning-based method, where the queue theory and the attention network are combined. Although there has been much work on the performance prediction for the GPS scheduling mechanism, limitations remain. Specifically, for the simplicity of performance prediction, a particular traffic model (e.g., short-range dependent (SRD) or long-range dependent (LRD)) has often been considered in existing methods. The traffic arriving from different application requests in the real IoT environment is heterogeneous, and only considering a particular traffic model will lead to the deviation of the GPS performance prediction. In addition, there is no unified benchmark in the open literature for fairly comparing existing methods.

**Figure 1:**
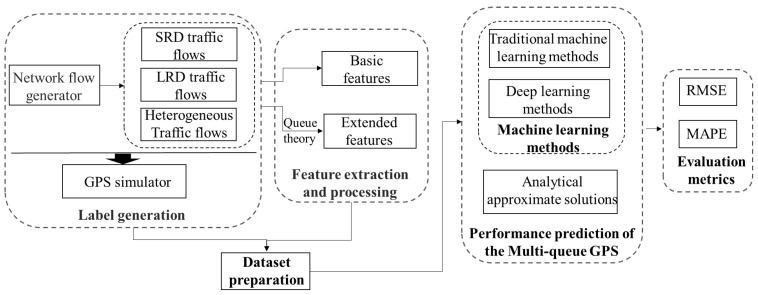
The overview framework of the benchmark.

**Figure 2:**
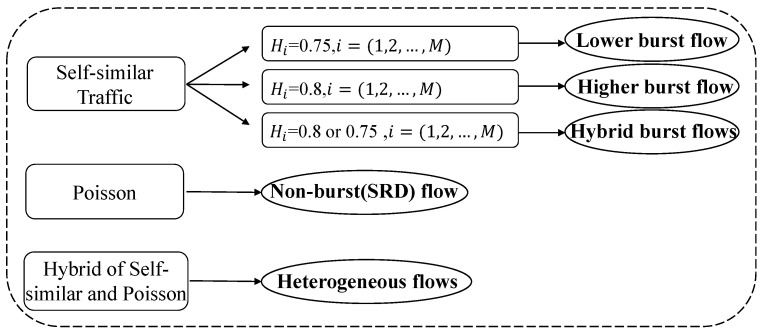
The five datasets.

To consider the heterogeneity of traffic models and explore the fair comparison of different performance prediction methods of GPS scheduling mechanisms, this paper innovatively designed an AI-enabled traffic-oriented benchmark (ToGPS), which is shown in Figure 1. To involve the heterogeneous network flow in the performance prediction of GPS, ToGPS combines two classical traffic models: the Poisson traffic model and the self-similar traffic model, which are proven to be representative traffic models for SRD and LRD traffic models, respectively. Based on the different combinations of the two kinds of traffic models, five types of datasets, including Lower burst flow, Higher burst flow, Hybrid burst flow, Non-burst flow, and Heterogeneous flow, have been designed, which are shown in Figure 2. In addition, for the fair comparison of different performance prediction methods of GPS, a unified dataset format and unified evaluation metrics have been designed. Specifically, the unified dataset was formed through a dataset preparation module, including a label generation module and a feature extraction and processing module. First, through the label generation module, labels for five datasets were generated using an improved GPS simulator, where the average queue length of each application service queue and the total queue length can be obtained. Then, the feature extraction and processing module reconstructed the final dataset format corresponding to different kinds of performance prediction methods of GPS. To clarify, the performance prediction methods of the GPS were divided into machine learning methods, deep learning methods, and analytical approximate solutions. Although the existing methods are only based on deep learning-based methods and approximate analytic methods, to conduct a more comprehensive exploration of the performance prediction methods, the benchmark fills the gap in the performance prediction of GPS in traditional machine learning methods, where classical traditional machine learning methods (i.e., decision tree and XGBoost) are included. Then, unified evaluation metrics were designed by combining the total queue length for the overall GPS scheduling system and the queue length for each application service queue, where the root-mean-square error (RMSE) and mean squared error (MAPE) were utilized to evaluate the accuracy of each performance metric for the application service. Based on the performance benchmark, extensive traffic-oriented experiments were conducted on five traffic datasets, and three types of performance prediction of the GPS and the results from a knowledge perspective and a data perspective were analyzed comprehensively. Through the experimental analysis, it is observed that the network traffic has a significant impact on the accuracy of different performance prediction methods for GPS. This variability is attributed to differences in traffic characteristics and the burstiness of the traffic. In addition, knowledge-driven information is effective in GPS performance prediction in terms of different server loads and traffic patterns. Deep learning-based methods, in particular, show promise when combined with knowledge-driven information.

The contributions of this paper can be summarized as follows:

- This paper is the first to provide a benchmark for the performance prediction of the multi-queue GPS scheduling in terms of different network flow characteristics. The benchmark makes it possible to compare different performance prediction methods under a consistent experimental environment and comparison metrics.
- The benchmark first combines traffic with different characteristics (i.e., LRD traffic and SRD traffic) to design five traffic datasets to involve traffic heterogeneity. Then, a unified dataset format and unified evaluation metrics are designed for fair comparison of the performance prediction of the GPS.
- This paper concludes the best-fit method considering different network flow characteristics and server loads.
- This paper further performs complex experimental analysis at both the feature level and method levels under different traffic flows. The experimental analysis shows that the combination of knowledge-driven information and machine learning technology contributes significantly to the performance prediction of the GPS.

The rest of the paper is organized as follows. Section 2 introduces related works. Section 3 details the motivations for the performance prediction benchmark. Then, the detailed benchmark system design is introduced in Section 4. Section 5 details the experiments. Section 6 covers discussions and the future work. Lastly, Section 7 concludes this paper.

## 2. Related Works

This section first introduces the related work on GPS scheduling performance prediction under the IoT resource allocation field, including traditional machine learning-based methods and deep learning-based methods. Then, related works on approximate analytic methods for the GPS performance prediction are shown.

### 2.1. Traditional Machine Learning-Based Methods

Traditional machine learning methods [13,14,15] refer to a class of machine learning algorithms that appeared earlier and were widely used before the rise of deep learning. It mainly learns patterns and rules from data to perform task prediction, classification and clustering, etc. These methods often rely on feature engineering, which manually extracts and selects appropriate features to represent the data. They have the advantages of strong interpretability, fast training, the ability to process small datasets, few hyperparameters, and feature engineering. While they also have the advantage of complex feature engineering, they feature poor generalization ability limitations in dealing with nonlinear relationships.

Traditional machine learning is widely used in resource allocation management in IoT networks [16,17], including resource scheduling and traffic classification. For instance, Junaid et al. [1] proposed a resource-efficient clustering framework for social IoT applications that performs geographic text clustering hierarchically without significantly reducing clustering quality. Chauhan et al. [18] studied the resource scheduling method based on the Q-learning algorithm for smart home applications. To dynamically adapt to various traffic in real-life scenarios, Chinchali et al. [19] proposed a reinforcement learning (RL)-based method for scheduling traffic to improve the network performance in resource allocation. In addition, Rjoub et al. [20] employed a machine learning method to schedule tasks in a cloud-assisted IoT network. In [20], multiple criteria were used to improve the network performance. As traffic classification plays a crucial role in resource allocation, many works have focused on machine learning-based methods for traffic classification. For example, Qiao et al. [21] combined a time window method and machine learning-based methods to identify traffic. Specifically, a time window method was used to analyze and extract features from various IoT application flows, and a support vector machine and back propagation neural network were employed to identify the network traffic. To study the performance of the machine learning-based classification methods, Perera et al. [22] compared multiple widely used machine-learning methods such as the naive Bayes net, random forest, and decision tree algorithm, where random forest and decision tree proved to be the best classifiers for classifying traffic. Though lots of work has been conducted in the resource allocation field, there is little literature that focuses on performance prediction of resource allocation utilizing machine learning. To this end, this work studies the performance of the machine learning methods in the performance prediction of resource allocation.

### 2.2. Deep Learning-Based Methods

Traditional machine learning algorithms have certain advantages and limitations in solving various problems, and it is very important to select an algorithm that is suitable for the nature of the problem and the characteristics of the data. In recent years, with the rise of deep learning methods [23,24,25], some problems have achieved better results using deep learning methods. Deep learning uses artificial neural network models to learn and represent complex data features, which is suitable for various tasks, such as image recognition, natural language processing, speech recognition, etc. Common deep learning methods include multilayer perceptions, convolutional neural networks [26], transformers, etc. Deep learning methods have significant advantages in dealing with large-scale complex data, thereby achieving high accuracy prediction and flexibility. However, these methods also present some challenges, such as the demand for large amounts of data and computational resources, as well as interpretability issues of the model used. In practice, deep learning methods are usually used in combination with traditional machine learning methods to give full play to their respective advantages. Zhang et al. [27] developed a deep reinforcement learning approach to optimize spectrum resource allocation, where a cooperative strategy between secondary users and primary users is used. Zhou et al. [28] proposed a deep learning-based framework for traffic prediction. In [28], the deep learning-based framework can adaptively choose the optimal model for traffic prediction, and internal relationships among traffic flow data can be extracted. To maximize the classification accuracy of the sensor data in IoT networks, Chun et al. [29] proposed a resource allocation scheme based on binarized neural networks, which utilizes wireless channel state information and data-driven methods to maximize the classification accuracy on the server side while meeting the total transmit power constraints. To solve the problem of resource allocation in the Internet of Things, ElHalawany [30] proposed two recursive neural network models. Through these models, IoT nodes can upload information to a nearby centralized gateway by reusing the communication channels of traditional cellular users, thus achieving the allocation of underlying IoT resources. To obtain accurate performance prediction for resource allocation on demand, Zhang et al. [12] designed a knowledge-driven multi-queue GPS performance prediction method, called DLPE, which manually selects relevant features and introduces them into the original features by analyzing the relevant theoretical knowledge of multi-queue GPS. In [12], the authors were the first to try to combine feature engineering traditional machine learning with a deep neural network.

### 2.3. Approximate Analytical Methods

For approximate analytical methods of performance prediction problems in on-demand resource allocation, much work has focused on the performance prediction of GPS scheduling systems. Zhang et al. [31,32] analyzed the asymptotic decay rate of the queue length tail distribution of two-queue and multi-queue systems under the GPS scheduling system, and the results were obtained by using the principle of large deviation of sample paths. Both works focused on the G/D/1 queue. Bertsimas et al. [33] obtained the lower and upper bounds of the asymptotic large deviation of each buffer overflow probability under a discrete-time GPS scheduling mechanism. Mannersalo et al. [10] proposed an empty buffer approximation (EBA) method for priority queues and a rough full link approximation method (RFLA)for the GPS scheduling mechanism to infer the queue length distribution (QLD). A multi-queue priority queue (PQ) scheduling system and a two-queue GPS scheduling system were studied. Jin and Min [34] proposed a new flow decomposition method for a PQ-GPS system, which decomposes the integrated system into three independent single-queue single-server systems. Their integrated system consists of a priority queue system and a two-queue GPS scheduling system. EBA is used to analyze the QLD of each subsystem. In addition, Ashour et al. [35] provided an analytical framework to evaluate the performance of multi-queue PQ systems, and they modeled the multi-queue as a two-queue system. At the same time, they also provided an analytical technique to infer the queue length of a multi-queue system under the principle of generalized processor sharing [36]. Although Ashour et al., have studied multi-queue PQ and multi-queue GPS, no general analysis result of the multi-queue GPS scheduling system has been obtained. multi-queue GPS mainly refers to a GPS system with more than two queues, and it is difficult to analyze its performance. Based on this, Zhang et al. [11] gave an approximate analytical solution of the queue length distribution for multi-queue GPS. In order to further improve the accuracy of the multi-queue GPS performance prediction results, Zhang et al. [12] designed a knowledge-driven performance prediction method based on a deep learning network by combining GPS theoretical analysis and deep learning.

Although many methods can be used to solve the problem of GPS performance prediction, such as traditional machine learning methods, deep learning methods, and approximate analytical methods, there is a lack of a benchmark for the unified comparison and analysis of GPS performance prediction methods. This is not conducive to the further improvement of the accuracy of multi-queue GPS performance prediction. Based on this, this paper aims to design a benchmark to provide a unified platform for evaluating various performance prediction methods.

## 3. Motivations

To clarify the motivations of this work, this section first emphasizes the significance of GPS scheduling, thus introducing a classic application under centralized resource allocation scenarios. Then, the importance of benchmarking is illustrated.

### 3.1. Application Scene

**Fairness guidance for the resource allocation:** The development of IoT technology promotes the growth of the number of access devices to the network. Plenty of devices such as smartphones, intelligence appliances, and smart cars access the network competing for network resources (i.e., communication resources and computation resources). Resource allocation is required to be efficient and fair for resource limitations. Efficiency means that the proper resource allocation can be achieved quickly and flexibly. Fairness means that each device can obtain a satisfactory quality of service. For the sake of fairness and flexible parameter settings, GPS can be regarded as an ideal scheduling mechanism guiding fair and efficient resource allocation.

### 3.2. Motivations for Performance Benchmarking

The performance prediction of multi-queue GPS has always been the focus of the research. However, no closed-form solutions can be found to obtain the exact performance prediction of each queue in the GPS, thereby limiting the application of GPS on fair resource allocation. In order to find an accurate solution for the performance prediction of each queue, a benchmark comparing the varieties of methods is of vital importance. Suitable methods can be selected that correspond to certain scenarios. Based on the optimization methods, the following targets can be achieved:

- To find out bottlenecks and gaps for improvement and whether the server capacity should be improved for the sake of the fairness objective.
- To provide fairness guidelines on the parameter configuration weights assigned to each flow network flow control.
- To propose and implement optimizations to improve performance.

## 4. Benchmark System Design

For clarity, this section begins with an introduction of the problem statement for the performance prediction of multi-queue GPS. Then, the design of the benchmark system is introduced, as shown in Figure 1. Firstly, the network flow preparation part is introduced to generate the network flows. Then, feature extraction and processing are employed to extract and process features. Following that, benchmark baselines are organized, which include machine learning-based methods and approximate analytical solutions for the performance prediction of multi-queue GPS. Lastly, RMSE and MAPE are utilized as evaluation metrics.

### 4.1. Problem Statement

This section intends to introduce the problem statement of the performance prediction of multi-queue GPS and the goal of this work. For the sake of understanding, Table 1 lists the definitions of the terminology.

**The performance prediction of the multi-queue GPS system:** Given a multi-queue GPS server with a server capacity *C* serving M(M≥1) flows, we assume that the arrival process of the flow obeys different traffic stochastic model *f*, where f∈{0,1} and 0 denote the Poisson traffic, and 1 denotes the self-similar traffic. Poisson traffic and self-similar traffic will be introduced in the flowing part. Then, we denote $P_{m}^{f}\left(t\right),t\in R$ as the cumulative arrival process of the network, where m∈{1,2,...,M}.

To allocate service capacities to different flows, each flow is assigned a fixed weight $r_{m}$, where $0<r_{m}<1$ and $\sum_{m=1}^{M}r_{k}=1$. Then, each flow $flow_{m}$ is guaranteed a minimum guaranteed service rate of $g_{m}=r_{m}C$, when it is served, based on the definition of the GPS mechanism referring to [37]. Waiting queues for each single flow, $SF=\{sf_{1},sf_{2},...,sf_{M}\}$, appear when the arrival rate exceeds the service rate, thereby referring to the single queue. The performance prediction of the multi-queue GPS system is to evaluate the performance of each single queue, such as average queue length $LP=\{lp_{1},lp_{2},...,lp_{M}\}$ and average queue delay $LD=\{ld_{1},ld_{2},...,ld_{M}\}$. Referring to the statement in [12], this problem is denoted as the multi-queue GPS problem.

**The goal:** The goal is to conduct an in-depth study of the performance prediction for the multi-queue GPS system, the learning-based methods—including traditional learning methods and deep learning methods—and the approximate analytical methods used on the multi-queue GPS system in terms of different flow characteristics are compared. Upon examining the experiment results, some observations aimed at analyzing the limitations of these methods and finding the proper methods to deal with the multi-queue GPS problem under a certain scenario are included.

### 4.2. Dataset Preparation

Network services put packets on the network link when waiting for the process of the server. Due to the uncertainty of the network service requirements, it is of great significance to accurately describe network flow characteristics. Extensive empirical and theoretical studies have been devoted to characterizing network traffic [38]. Various network flow models have been studied, which can be divided into two types: the short-range dependence traffic model (SRD) and the long-range dependence traffic model (LRD). Different network flow models focus on different characteristics of the network flow. As the classical traffic models, the Poisson process [39], and self-similar process [40,41], which are the representative traffic models for the SRD and LRD, respectively, play a vital role in characterizing traffic behaviors. This work mainly explores the effect of the Poisson process, the self-similar process, and the heterogeneous traffic comprising the Poisson process and the self-similar process on the performance prediction of the multi-queue GPS system. The following will introduce the Poisson traffic and the self-similar traffic.

**Poisson traffic flow:** The Poisson model can well meet the early network modeling requirements and has played a great role in network design, maintenance, management, and performance analysis [42]. Some researchers claim that the Poisson model may fail to characterize the present network flow [43]. Others argue that current network traffic can also well characterize the Poisson model in terms of subsecond time scales [44]. Assume that packets are distributed independently when they arrive and are only related to a single rate parameter. If the number of packets arriving in the time series $\left[t_{1},t_{2}\right]$ conforms to the Poisson distribution with parameter $A_{m}^{0}*\left(t_{2}-t_{1}\right)$, the arrival process of the packet is called a Poisson process, where $A_{m}^{0}$ is the arrival rate of the Poisson flow, and the number of the flow is *m*. Then, based on the Poisson process, the probability of the arrival of *n* packets within time $\left[t_{1},t_{2}\right]$ can be expressed through Equation (1). In Equation (1), $P_{m}^{0}\left(t_{1}\right)$ and $P_{m}^{0}\left(t_{2}\right)$ denote the cumulative arrival amount at time $t_{1}$ and $t_{2}$, respectively, for the $flow_{m}$, and the flow obeys a Poisson process.

(1) $$\begin{array}{cc} \; & Pr\left[\left(P_{m}^{0}\left(t_{2}\right)-P_{m}^{0}\left(t_{1}\right)\right)=n\right]=\frac{e^{-A_{m}^{0}\left(t_{2}-t_{1}\right)}{\left(A_{m}^{0}\left(t_{2}-t_{1}\right)\right)}^{n}}{n!}\\ & \;n=0,1,\ldots \end{array}$$

**Self-similar traffic flow:** With the development of network technology, the data carried by network traffic present diversity (such as text, images, video, real-time services, etc.), which makes network traffic characteristics more complex and bursty. Existing studies have found that the network flow shows self-similarity through studies on the flow analysis of the local area network, the IP business flow of the World Wide Web, and the VBR video business flow [40,45,46]. Fractal Brownian motion has proved to be the most efficient approach for modeling self-similar traffic, which is called fractal Brownian network flow.

Denote the cumulative arrival process of the self-similar traffic by $P_{m}^{1}\left(t\right),t\in R$, where $P_{m}^{1}\left(t\right)=A_{m}^{1}t+Y_{m}\left(t\right)$, and the amount of $flow_{m}$ arrives in an interval of $\left[t_{1},t_{2}\right]$ by $\alpha_{m}\left(t_{1},t_{2}\right)=P_{m}^{1}\left(t\right)-P_{m}^{1}\left(s\right)$. $A_{m}^{1}$ is the mean arrival rate, and $Y_{m}\left(t\right)=\sqrt{a_{m}A_{m}^{1}{\bar{Z}}_{m}\left(t\right)}$. Here, $a_{m}$ is the variance coefficient of $\beta_{m}\left(t\right)$, and ${\bar{Z}}_{m}\left(t\right)$ is a centered fBm with the variance function ${\bar{v}}_{m}\left(t\right)=t^{2H_{m}}$, where $H_{m}\in \left[0.5,1\right]$ is the physically significant range of the Hurst parameter, which indicates the degree of self-similarity [47] and the long-range dependence. Then, the variance function of $\alpha_{m}\left(t\right)$ can be given by the following:

(2) $$Var\left\{\alpha_{m}\left(t\right)\right\}=Var\left\{Y_{m}\left(t\right)\right\}=a_{k}A_{m}^{1}{\bar{v}}_{m}\left(t\right)=a_{m}A_{m}^{1}t^{2H_{m}}$$

#### 4.2.1. Label Generation

**Dataset classification based on network flow models:** In order to study the effect of the performance prediction methods for the multi-queue GPS system, a GPS simulator was utilized to obtain the performance of each queue. Five datasets were obtained, as are shown in Figure 2. To study the effect of the different network flows on the performance prediction of the multi-queue GPS system, these datasets were derived in terms of different network flow models. As Figure 2 shows, the first three datasets are based on self-similar traffic considering different Hurst parameters. H=0.75 and H=0.8 were chosen as examples, for they have proven to be more efficient in the modeling of modern network traffic [48]. The five datasets are introduced in detail as follows:

- Dataset **Lower burst flow**: Each flow obeys the self-similar process, and the Hurst parameter of each flow is set as H=0.75;
- Dataset **Higher burst flow**: Each flow obeys the self-similar process, and the Hurst parameter of each flow is set as H=0.8;
- Dataset **Hybrid burst flows**: Each flow obeys the self-similar process, and each flow is set with different Hurst parameters.
- Dataset **Non-burst (SRD) flow**: Each flow in the multi-queue GPS that has arrived obeys the Poisson process.
- Dataset **Heterogeneous flows**: Some flows have arrived obeying the Poisson, and others obey the self-similar process.

**Figure 3:**
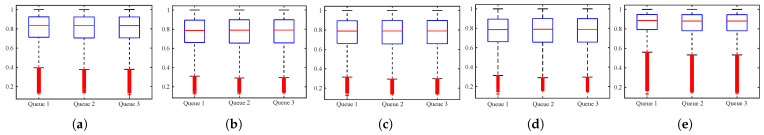
The distribution of the utility of each flow in five datasets. (**a**) Lower burst flow; (**b**) Higher burst flow; (**c**) Hybrid burst flows; (**d**) Heterogeneous flows; (**e**) Non-burst flow.

**Figure 4:**
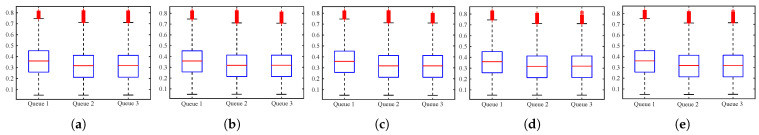
The distribution of the proportion of the arrival rate of each flow in five datasets. (**a**) Lower burst flow; (**b**) Higher burst flow; (**c**) Hybrid burst flows; (**d**) Heterogeneous flows; (**e**) Non-burst flow.

**Figure 5:**
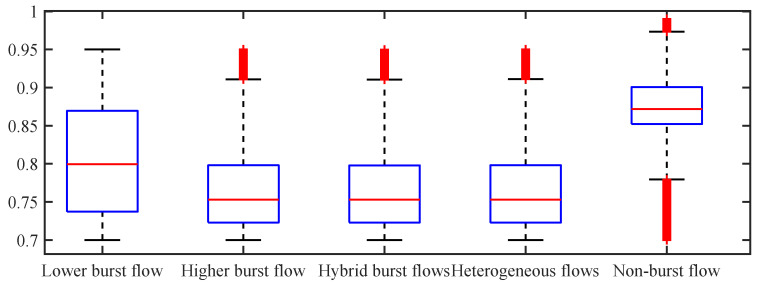
The distribution of the utility.

**The distribution of the dataset:** To make the setup of the platform transparent and prove the effectiveness and applicability of the dataset selection, the distribution of these five datasets is mainly introduced in the following. The data distribution of these five datasets is shown in Figure 3, Figure 4 and Figure 5. Without a loss of generality, three-queue GPS has been mainly considered. Figure 3 shows the server utilization distribution of each flow on the five datasets, where the server utilization is calculated according to the guaranteed service capacity of each flow, that is, $\rho_{m}=A_{m}^{f}/g_{m}$. Figure 3 shows that in each dataset, the distribution of the server utilization of the three network flows has similarity, including upper bound, lower bound, median, upper quartile, and lower quartile. There are great differences among the datasets.

Figure 4 shows the distribution of the proportion of the arrival rate of each network flow in the five datasets, that is, $pr_{m}=A_{m}^{f}/\sum_{k=1}^{M}A_{k}^{f}$. As shown in Figure 4, the distribution of the proportion of arrival rates of the corresponding network flows among the datasets is similar, while the distribution of each network flow in the same dataset has certain differences.

Figure 5 shows the distribution of the overall server utilization, where the overall server utilization is the server-based service capacity, which is $A_{m}^{f}/C$. As shown in Figure 5, the server utilization of the selected dataset is consistent with the characteristics of network flows. For example, due to the burstiness of self-similar network flows, the server utilization of self-similar network flows was smaller than that of non-burst flows as a whole.

**Multi-queue GPS simulator:** The inputs of the GPS simulator are various network flow sequences generated according to the network flow model, and the output is the queue performance metric of the corresponding queue of the network flow processed by the GPS scheduling mechanism, such as queue length. The principle of the multi-queue GPS simulator is detailed in the following. Suppose that *M* network flows arrive; the server has a service capacity of *C*, and the weights are $\left\{r_{1},r_{2},\ldots ,r_{m},r_{M}\right\}$. When only one network flow $flow_{m},m\in \left(1,M\right)$ arrives at the requested service, the server allocates all its service capacity to $flow_{m}$. When any two network flows arrive with $flow_{m}$ and $flow_{k},m,k\in \left(1,M\right)$, the server allocates service resources to these two flows according to their relative weights, namely $flow_{m}$. The service capacity obtained is $\frac{r_{m}}{r_{m}+r_{k}}C$, that is, the service capacity obtained by $flow_{k}$ is $\frac{r_{k}}{r_{m}+r_{k}}C$; similarly, when more than two network flows arrive, each network flow obtains the service capacity resource according to its relative weight.

#### 4.2.2. Feature Extraction and Processing

In order to achieve the fairness and reliability of the benchmark platform, the input features were normalized and unified. The input features were divided into basic features and extended features. Basic features mean features obtained through the general statistical analysis (i.e., average value and variance) in terms of the network flow and the GPS’s definition. Extended features denote knowledge-driven features based on in-depth theory analysis (i.e., queue theory).

**Basic features:** The basic features (BFs) were divided into two parts: network flow-related and the GPS server-related. Considering the effect on the quality of the service (QoS) and the network flow model mentioned above, the network flow-related features included the mean arrival rate, the variance of the arrival of the flow, and the packet length, which can be denoted as $NF=\left(Arr,Var,Pl\right)$, where $Arr\{A_{1}^{f},A_{2}^{f},\ldots ,A_{M}^{f}\}$ denotes the vector of the mean arrival rate. Based on the definition of GPS, the GPS server-related basic features can be denoted as $GPS_{S}=\left(W,C\right)$, where *W* denotes the weight assigned to each flow, and *C* denotes the resource capacity that the GPS server can provide.

**Extended features:** Based on the queue theory, the basic features can be extended to capture better effective features that affect GPS performance. Referring to Jin’s work [49], the extended features (EFs) mainly include the minimum guaranteed capacity MU, the proportion of the arrival rate PA, and the utility based on the minimum guaranteed capacity UM for each flow feeding into the GPS server, which can be denoted as EF=(MU,PA,UM).

Therefore, the knowledge-driven features comprise basic features and the extended knowledge features, which can be denoted as IF=(NF,EF).

### 4.3. Performance Prediction of the Multi-Queue GPS System in the Benchnmark

This section illustrates the variety of performance prediction methods for multi-queue GPS employed in the benchmark. Traditional approximate analytical methods, traditional machine learning-based methods, and deep learning-based methods are three different types of methods that can conduct performance prediction for GPS scheduling. Existing methods mainly predict the performance metrics using traditional approximate analytical methods and deep learning-based methods. For example, Mannersalo et al. [9] employed large deviation principle-based (LDPs) analytical methods to derive the total queue length distribution for overall GPS scheduling. In [11], the authors use empty buffer approximate (EBA)-based analytical methods to derive the queue length distribution of each application service. Due to the limitations of analytical methods, only approximate solutions for performance prediction can be obtained. To obtain accurate performance metrics, Zhang et al. [12] predicted the performance of the GPS scheduling mechanism by employing a knowledge-driven deep learning-based method, where queue theory and the attention network are combined. This marked a successful endeavor in leveraging deep learning-based methods for GPS performance prediction, thereby highlighting the considerable potential in this domain. However, it is essential to recognize that the application of deep learning-based methods necessitates an extensive dataset and prolonged training periods, thus making them less viable in situations where datasets are scarce and time constraints are high. While there is no research on the application of traditional machine learning with respect to the performance prediction of the GPS scheduling mechanism, it serves as a viable alternative method for its excellent performance of machine learning methods in handling small datasets and time constraints. In addition, traditional machine learning has demonstrated strong performance in network resource allocation [22] To undertake a more exhaustive investigation of the performance prediction methods for GPS, the AI-enabled benchmark incorporates classical traditional machine learning methods, specifically decision tree and XGBoost. To clarify, the deep learning-based methods for the performance prediction of GPS have been further divided into deep learning methods without knowledge-driven information and the knowledge-driven deep learning method in the benchmark. Three types of methods are introduced as follows.

#### 4.3.1. Traditional Machine Learning Method

**Decision_CART:** Decision trees are a supervised learning method that can be used to deal with classification problems or regression problems. As a relatively effective and powerful traditional machine learning method, the benchmark of this paper takes it as one of the baselines to solve the GPS performance prediction problem. Specifically, the classification and regression tree (CART) algorithm [50] uses a binary tree to simplify the size of the decision tree, which can improve the efficiency of decision tree generation. Therefore, the decision tree implemented by the CART has been utilized in the benchmark, and it is referred to as Decision_GBRT.

**Boosting_Xgboost:** When weak models are combined correctly, more accurate and/or robust models can be obtained. The integration algorithm is exactly the algorithm that can improve the efficiency of the machine learning model. Specifically, boosting, which is one of the integration algorithms, learns these weak learners sequentially (each base model depends on the one before it) in a highly adaptive way, and it combines them according to some deterministic strategy. As demonstrated in Xgboost [51,52], one of the implementations of the boosting introduces a L2 regularization term of the leaf weight, which is conducive to the model to obtain a lower variance. Xgboost is utilized for implementation, which we refer to as Boosting_Xgboost.

#### 4.3.2. Deep Learning Method without Knowledge-Driven Information

As a subdomain of machine learning, unlike traditional deep learning methods, deep learning methods can automatically extract advanced features without the need for artificial feature engineering [53].

**MLP:** Multilayer perception (MLP) [54] is the basic deep learning method that simply concludes the input layer, hidden layer, and output layer. The different layers of MLP neural networks are fully connected.

**GCN:** In order to deal with more complex data structures, graph convolutional networks (GCNs) [55] have attracted the attention of researchers. The GCN has designed an elegant method to extract features from graph data so that these features can be used to produce the node classification, graph classification, and link prediction of graph data. In addition, graph embedding can be obtained by using a GCN. Considering the tightly coupled relations among queues in GPS, the input features of each queue and the server can be organized as graph data.

#### 4.3.3. Knowledge-Driven Deep Learning Method

Zhang et al. [12] proposed a knowledge-driven deep learning method for the performance prediction of the multi-queue GPS system called the DLPE. The input features of the DLPE are the combining of the basic features and the extended knowledge-driven features. In addition, an innovative fused model considering knowledge-driven information is designed to dig out in-depth relations for each queue of the multi-queue GPS system.

#### 4.3.4. Analytical Approximate Methods

Though it is significantly hard to derive the performance of each queue in the multi-queue GPS system by utilizing the analytical methods, the approximate analytical methods have played a vital role in the performance prediction of the multi-queue GPS system. In this benchmark, the approximate analytical methods for the total queue (MPPA) [9] and for each queue (EBA_Multi) [11] have been compared. In terms of the complicated relationship among the queues and the server, only the upper and lower bounds can be devised exactly. For uniform comparison, this benchmark uses the geometric mean of the upper and lower bounds as the maximum performance value [56].

To clarify the approximate analytical methods, the determined function is introduced first, as shown in Equation (3). In Equation (3), $l_{x}$ is the queue length at time *t*, and $v_{m}\left(t\right)$ is the variance function of $flow_{m}$.

(3) $$\chi \left(t\right)=\frac{{\left(-l_{x}+\left(C-\sum_{m=1}^{M}\theta_{m}\right)t\right)}^{2}}{\sum_{m=1}^{M}v_{m}\left(t\right)}$$

**MPPA:** Based on the most probable path method, the probability of the queue length $l_{x}$ can be obtained using Equation (4). The MPPA mainly focuses on the total queue of the GPS where the aggregate flows are considered.

(4) $$\frac{\exp\left(-\frac{1}{2}\chi \left(t_{l_{x}}\right)\right)}{\sqrt{2\pi {\left(1+\sqrt{\chi \left(t_{l_{x}}\right)}\right)}^{2}}}\leq P\left(L_{x}>l_{x}\right)\leq \exp\left(-\frac{1}{2}\chi \left(t_{l_{x}}\right)\right)$$

**EBA_Multi:** To obtain the queue length of each queue in the multi-queue GPS system, Zhang et al. [11] further utilized the empty buffer approximate (EBA) method to estimate the service capability actually obtained $cap=\left(c_{1},c_{2},...,c_{M}\right)$ as the probability of the queue length of each queue. We substitute M=1 into Equation (3); then, the determining function and the bounds for each queue can be obtained by referring to Equations (3) and (4), respectively. Equation (5) is the determining function for each queue.

(5) $$\chi \left(t\right)=\frac{{\left(-l_{x}+\left(c_{m}-\theta_{m}\right)t\right)}^{2}}{v_{m}\left(t\right)}$$

### 4.4. Evaluation Metrics

In order to evaluate the effect of each method on the GPS performance prediction, the benchmark uses the standard regression evaluation indicators root-mean-square error (RMSE) and mean absolute percentage error (MAPE) to compare the performance of each method. Different queue metrics provide information about different aspects of the network. The total queue length for all application services is utilized to reflect the overall congestion situation of the scheduling system to locate the congested server or cloud service center and the congestion. The queue length for each application service is utilized to reflect each application requesting the server. By monitoring the queue length of each application, the resource allocation weight of the application is adjusted appropriately, or the flow of the application is limited to relieve congestion.

## 5. Experiments

In order to evaluate the effect of each performance prediction method for the multi-queue GPS system, extensive traffic-oriented experiments have been conducted on the benchmark.

### 5.1. Experiment Settings

To dig out the in-depth effect of different methods on the performance prediction of the GPS scheduling, two traffic-oriented experiments were conducted as follows.

**First**, to comprehensively explore the performance of each performance prediction method considering different traffic patterns and different utilization loads, experiments were conducted on five kinds of traffic datasets and three kinds of performance prediction methods, which are shown in Section 4.3. Specifically, five datasets were generated based on different traffic models, including Lower burst flow, Higher burst flow, Hybrid burst flows, Non-burst flow, and Heterogeneous flows, as shown in Section 4.2.1. Three-queue GPS has been considered here. Each of the five datasets was divided into three parts based on the server’s utilization load, which is denoted as utility. As nearly no queues exist under the 70% utility of the server and the server becomes heavily congested over 95%, the utilities from 70% to 95% were chosen. To refine the server utilization, the interval was divided into three segments: server utility ranges from 70% to 80%, denoted as Ut=(0.7,0.8); server utility ranges from 80% to 90%, denoted as Ut=(0.8,0.9); and server utility ranges from 90% to 95%, denoted as Ut=(0.9,0.95). Then, the input data format of the five datasets was set to correspond to different performance prediction methods. As shown in Section 4.1, machine learning-based methods (i.e., Desision_CART and Boosting_Xgboost), deep learning-based methods without knowledge-driven information (i.e., MLP, MLP1_Norm, and GCN) and analytical approximate methods were fed with datasets formatted with the basic features. The knowledge-driven deep learning methods (i.e., the DLPE) were fed with datasets formatted with the knowledge-driven features.

**Second**, to further explore the effect of the knowledge-driven feature on deep learning-based methods, experiments were conducted on the five datasets and deep learning-based methods without knowledge-driven information. Each dataset was also divided into three parts, which are Ut=(0.7,0.8), Ut=(0.8,0.9), and Ut=(0.9,0.95). In this scenario, knowledge-driven features inspired by the DLPE were added as the input features of the deep learning-based methods without knowledge-driven information.

### 5.2. Experiment Results and Analysis

In this section, the experiment results and analysis are presented in three aspects: the effect of the performance prediction methods under different server loads, the comparison of the methods with and without knowledge-driven information, and the comparison of the learning-based methods and the approximate analytical methods, where four observations were obtained.

#### 5.2.1. The Effect of Performance Prediction Methods under Different Server Loads

**Figure 6:**
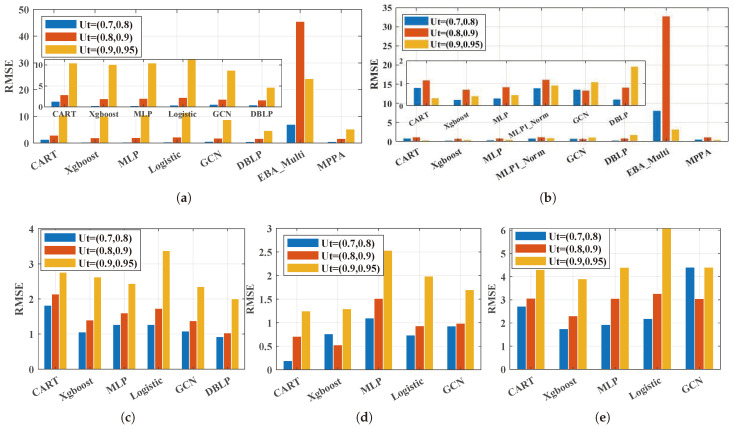
The RMSE of the total queue in GPS. (**a**) Lower burst flow; (**b**) Higher burst flow; (**c**) Hybrid burst flows; (**d**) Heterogeneous flows; (**e**) Non-burst flow.

**Figure 7:**
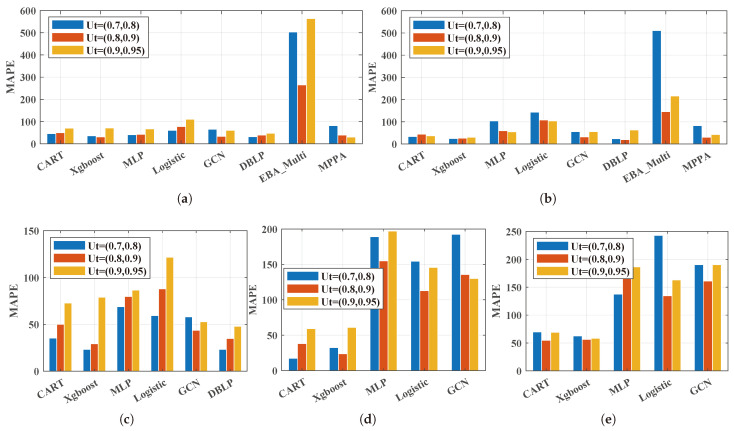
The MAPE of the total queue in GPS. (**a**) Lower burst flow; (**b**) Higher burst flow; (**c**) Hybrid burst flows; (**d**) Heterogeneous flows; (**e**) Non-burst flow.

This section first analyzes the effect of multiple multi-queue GPS performance prediction methods under different network traffic scenarios in detail from the perspective of the GPS queue, average queue, and each subqueue, as shown in Table 2, Table 3, Table 4, Table 5 and Table 6. Secondly, by further analyzing the effect of each prediction method from the perspective of total queue prediction, several observations were summarized, as are shown in Figure 6 and Figure 7.

Firstly, Table 2, Table 3, Table 4, Table 5 and Table 6 show the performance comparison of the multi-queue GPS performance prediction methods under different network flow characteristics such as burst network flow (LRD), non-burst network flow (SRD), and heterogeneous network flows. Burst network flows were further divided into lower burst network flows, higher burst network flows, and hybrid network flows.

Table 2, Table 3 and Table 4 show the performance of each performance prediction method under burstiness (LRD) network traffic. Since the method of the MAPE can only predict the GPS queue, but not the performance of each single queue and only for the case that each network flow has the same burstiness, only the RMSE and MAPE of the MPPA predicted results on the total GPS queue are shown in Table 2 and Table 3. And the prediction results for the MPPA are not shown in Table 4. Given that the DLPE is the performance prediction method for burst (LRD) network flows, it can be seen from Table 2, Table 3 and Table 4 that the DLPE performed best in most cases in terms of the GPS total queue and average queue. However, in a few cases, the DLPE did not perform optimally in terms of the GPS total queue and average queue. Except for the single queue prediction, the effect was better than the other prediction methods. For example, in Table 2, when the utility was Ut=(0.7,0.8), the DLPE predicted that the RMSE values of the total queue and the average queue were not the smallest, but the RMSE of queue 1 reached the optimal and was reduced by 19.35% compared to the suboptimal Boosting_Xgboost. In addition, when the utility was Ut=(0.8,0.9), the DLPE predicted that the MAPE values of the total queue and the average queue were not the minimums, while the MAPE values of queue 1 and queue 2 were both the minimums. The same findings can be observed in Table 3 and Table 4. Thus, it can be concluded that the DLPE, which considers the knowledge information related to bursty network flows, has a great advantage in predicting the performance of GPS for bursty network flows. No matter from the perspective of GPS queue, average queue, or single queue, similar conclusions can be obtained.

Table 5 and Table 6 show the prediction effects of each GPS prediction method under non-burst network traffic and heterogeneous network traffic, respectively. Since there is no accurate prediction method for non-burst network flow and heterogeneous network flow in the open literature, only the common traditional machine learning algorithms and deep learning algorithms were compared here. It can be seen that traditional machine learning methods seemed to demonstrate better prediction results compared to poorly designed deep learning methods. Specifically, Boosting_Xgboost showed better prediction results on non-burst network flows in most cases. However, Decision_CART showed its performance advantage on heterogeneous network flows. It can be inferred that when the deep neural network does not have a well-designed structure and knowledge-driven new assistance, its prediction effect on GPS performance prediction is not as good as the traditional machine learning method.

Secondly, to further analyze the prediction effect of each prediction method, Figure 6 and Figure 7 show the bar charts of the comparison of different performance prediction methods for the total queue length under different server utilizations. Based on the experimental results, the following observations were made:

**Observation 1:***The well-designed knowledge-driven deep learning methods showed excellent performance on the performance prediction of the multi-queue GPS system under lower utility ranges.* As the existing well-designed knowledge-driven method, which is denoted as DBLP, is designed for self-similar traffic, only datasets **Lower burst flow**, **High burst flow**, and **Hybrid burst flows** are analyzed here. As is shown in Figure 6a–c, the DBLP always performed well under utility (0.7, 0.8) and utility (0.8, 0.9). However, under utility (0.9, 0.95), the DBLP failed to perform well under some scenarios such as **Higher burst flow** and **Hybrid burst flows**. This may be because the percentage of the high utility ranges in the dataset is rather smaller than the lower utility, which can be seen in Figure 5. In Figure 6, the same results can be found for dataset **Lower burst flow**, dataset **Higher burst flow**, and dataset **Hybrid burst flows**; the DBLP showed the lower MAPE under most utility ranges than the other performance predictions.

**Observation 2:***Deep learning methods may fail to work compared to the traditional machine learning methods for multi-queue GPS’s performance prediction.* As can be seen in Figure 6d,e and Figure 7d,e, for dataset **Heterogeneous flows** and dataset **Non-burst flow**, the traditional machine learning methods CART and Xbboost showed lower RMSE and MAPE values than the deep learning methods under most utility rages. Specifically, Xbboost always had the best performance in terms of the RMSE on dataset **Non-burst flow** under the three utility ranges. For the MAPE, Xbboost also had a great performance. While for dataset **Heterogeneous flows**, CART performed better on both the RMSE and MAPE under most scenarios. Similar findings can also be noticed on the datasets **Lower burst flow**, **Higher burst flow**, and **Hybrid burst flows** when the DLPE was not taken into consideration.

Based on this finding, why deep machine learning fails in this way can be explained reasonably. Firstly, traditional machine learning methods have strong power on feature acquirement and feature extraction, which enables the CART and Xbboost to show excellent performance on the performance prediction of multi-queue GPS. Secondly, though deep learning methods are famous for their powerful processing capability for complex problems, many training parameters may lead to training bias and susceptibility to noise. Thus, a well-designed structure for deep learning is necessary. MLP, Logistic, and the GCN are not well designed for focusing on the performance prediction of the multi-queue GPS system, which causes the failure.

#### 5.2.2. The Comparison of the Methods with and without Knowledge-Driven Information

So what can contribute to the performance improvement of the performance prediction of the multi-queue GPS system in terms of the deep learning methods? In order to clarify this question, the main part of the DBLP needs to be reviewed. The DBLP creatively introduces queuing theory-based knowledge-driven information leading to significant improvement when considering all the utilities. First, the knowledge-driven features are added as the input features. Then, a fusion model considering the queue theory is designed to capture the in-depth characteristics of each queue. In a word, knowledge-driven information plays a vital role in the performance improvement of the multi-queue GPS’s performance prediction. Therefore, it can be easily concluded that the role of knowledge-driven information is that the knowledge-driven features contribute to the improvement of the performance for the performance prediction of the multi-queue GPS methods employing deep learning methods. This will be verified in this part.

In order to verify that the knowledge-driven features play a significant role, the deep learning methods with and without knowledge-driven information were compared. MLP, Logistic, and the GCN were utilized as the base methods. Then, referring to the knowledge-driven features designed for the DBLP, the knowledge-driven features were added as the input features for MLP, Logistic, and the GCN to obtain the comparison methods. Through the experiment results, the following observation can be found.

**Figure 8:**
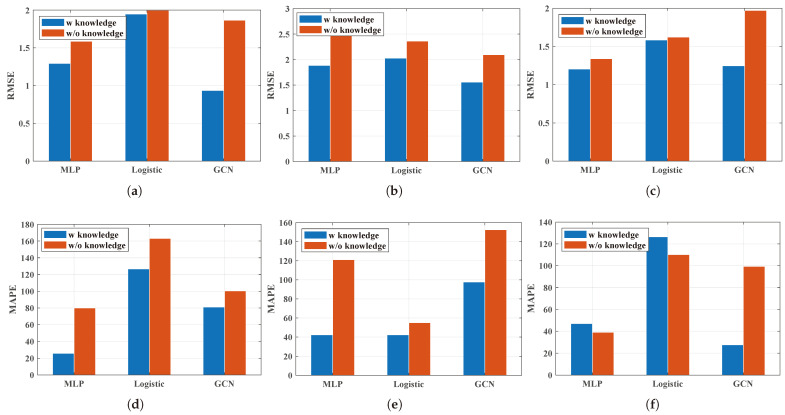
Comparison of methods with and without knowledge-driven information. (**a**) Lower burst flow; (**b**) Higher burst flow; (**c**) Hybrid burst flows; (**d**) Lower burst flow; (**e**) Higher burst flow; (**f**) Hybrid burst flows.

**Observation 3:***Knowledge-driven information does not work under the Hybrid burst flows.* Figure 8 compares the deep learning methods with the basic features as the input features and the deep learning methods considering the knowledge-driven features. MLP, Logistic, and the GCN were taken into consideration here. It can be noticed that for the RMSE, the methods considering the knowledge-driven features all performed better than the methods without knowledge-driven features. In addition, the methods considering the knowledge-driven features showed better performance than the methods without knowledge-driven features in most scenarios. For the dataset **Hybrid burst flows**, the MAPE of the MLP considering the knowledge-driven features performances was not so good. The reason can be easily explained. The knowledge-driven information is extracted from the queue theory on the GPS subject to the self-similar traffic with the same Hurst parameter. In contrast, the Hurst parameters in dataset **Hybrid burst flows** are different for each flow. Thus, the knowledge-driven information may not be so correct where noise may be introduced into the model, thereby resulting in training bias.

#### 5.2.3. The Comparison of the Learning-Based Methods and the Approximate Analytical Methods

**Observation 4:***Learning-based methods show better performance than the approximate analytical method for the performance prediction of the multi-queue GPS system in terms of each queue.* It can be seen from Figure 6 and Figure 7 that the EBA_Multi method showed large peak fluctuations. This may be because the EBA_Multi method performs better when not all of the queues’ guaranteed service capacity is larger than the arrival rate [11]. While the MPPA showed a small fluctuation, the MPPA focused on the total queue of the multi-queue GPS system. It has been proven that the MPPA method can predict the total queue of the multi-queue GPS system accurately. As the learning-based methods are data-driven methods, in theory, given enough data, machine learning can achieve a perfect fit. When a large amount of simulation data is obtained, the learning-based methods can easily perform better than the approximate analytical method.

## 6. Discussions and Future Work

In this section, valuable insights for the research of GPS performance prediction methods based on experimental analysis are summarized, and future work based on these insights is presented. Our observations indicate that the network traffic has a significant impact on the accuracy of different performance prediction methods for GPS. This variability is attributed to differences in traffic characteristics and the burstiness of the traffic. It is evident that the high burstiness and heterogeneity of the traffic contribute to an unbalanced distribution of the dataset, thus making performance prediction more challenging. To address this issue in future GPS performance prediction, attention should be given to preprocessing the dataset and finding viable methods to address dataset imbalance, such as dataset expansion or the resampling of minority class samples. Furthermore, we have found that knowledge-driven information is effective in GPS performance prediction. Deep learning-based methods, in particular, show promise when combined with knowledge-driven information. To maximize the impact of knowledge information on improving deep learning performance for GPS performance prediction, advancements in related theories should be pursued, and prior knowledge should be actively applied in diverse ways, including transfer learning, reinforcement learning, and data augmentation. Moreover, learning-based methods consistently demonstrated exceptional performance across all individual queues, whereas approximate analytical methods exhibited strong performance in certain cases when considering the total queue. Given the favorable performance of approximate analytical solutions in specific instances, there is potential to delve deeper into the theory of these solutions and enhance the performance of deep learning methods. For instance, integrating approximate analytical solutions into the optimization process of neural network learning could yield substantial improvements.

## 7. Conclusions

In order to evaluate the effect of different performance prediction methods on the multi-queue GPS system, this paper designed a benchmark in a unified way. This benchmark provides a way for comparing traditional machine learning methods, deep learning methods and analytical approximate methods in terms of different network flow characteristics. Through extensive experiments based on the benchmark, It was found that knowledge-driven information plays a vital role in the improvement of the performance of the deep learning methods for the performance prediction of the multi-queue GPS system. First, traditional machine learning methods perform better than deep learning methods in most scenarios when knowledge-driven information is not considered. Then, knowledge-driven feature construction can improve the performance of the deep learning methods.

## Figures and Tables

**Table 1:** The definition of the terminology.

Terminology	Definition
*C*	The server capacity of the multi-queue GPS system
*M*	The number of the traffic flow
$flow_{m}$	The *m*th traffic flow served by the multi-queue GPS system
*f*	The type of traffic stochastic model, where f=0 denotes the Poisson process, and f=1 denotes the self-similar traffic
$P_{m}^{f}\left(t\right)$	The cumulative arrival process of the traffic $flow_{m}$ until time *t* and flow_m obey *f*
$r_{m}$	The weight assigned to $flow_{m}$
$g_{m}$	The guaranteed service rate of $flow_{m}$
LP	The vector of the average queue length of each traffic flow
$lp_{m}$	The average queue length of $flow_{m}$
LD	The vector of the average queue delay of each traffic flow
$ld_{m}$	The average queue delay of $flow_{m}$
$A_{m}^{f}$	The arrival rate of $flow_{m}$, where $flow_{m}$ obeys the stochastic model *f*
$H_{m}$	The Hurst parameter of $flow_{m}$
NF	The flow-related features
Arr	The vector of the mean arrival rate
$GPS_{S}$	The server-related features
EF	The extended features
IF	The input features of the performance prediction

**Table 2:** Performance comparison of different methods for the performance prediction of GPS under lower burst traffic.

Utility of the Server	Methods	Decision_CART		Boosting_Xgboost		MLP		MLP1_Norm		GCN		DLPE		EBA_Multi		MPPA	
	Metrics	RMSE	MAPE	RMSE	MAPE	RMSE	MAPE	RMSE	MAPE	RMSE	MAPE	RMSE	MAPE	RMSE	MAPE	RMSE	MAPE
Ut = (0.7, 0.8)	GPS queue	1.363	45.271	**0.322**	36.077	0.326	40.635	0.430	60.215	0.610	65.425	0.510	**31.757**	6.965	503.397	0.524	81.482
	Average queue	0.454	15.090	**0.107**	12.026	0.109	13.545	0.143	20.072	0.203	21.808	0.170	**10.586 **	2.322	167.799	–	-
	Queue 1	0.192	13.202	0.093	10.862	0.123	17.539	0.129	17.639	0.132	16.117	0.075	7.107	2.465	133.338	-	-
	Queue 2	0.751	18.023	0.114	12.722	0.103	13.292	0.152	21.647	0.222	23.046	0.223	9.349	2.528	180.288	–	–
	Queue 3	0.420	14.047	0.115	12.493	0.100	9.804	0.149	20.929	0.256	26.262	0.212	15.301	1.972	189.771	–	–
Ut = (0.8, 0.9)	GPS queue	2.931	50.022	1.943	**31.019**	2.048	42.268	2.251	77.182	1.817	33.534	**1.669**	39.024	45.525	265.149	1.677	39.126
	Average queue	0.977	16.674	0.648	**10.340**	0.683	14.089	0.750	25.727	0.606	11.178	**0.556**	13.008	15.175	88.383	–	–
	Queue 1	0.551	14.041	0.478	9.195	0.497	19.987	0.580	24.513	0.460	8.651	0.386	8.221	29.014	75.945	–	–
	Queue 2	1.324	17.874	0.740	10.679	0.766	11.134	0.837	26.828	0.728	12.689	0.696	8.057	7.892	93.552	–	–
	Queue 3	1.056	18.107	0.725	11.145	0.785	11.147	0.834	25.841	0.629	12.194	0.587	22.746	8.619	95.651	–	–
Ut = (0.9, 0.95)	GPS queue	10.483	70.283	10.123	70.770	10.495	67.321	11.367	110.224	8.771	60.211	**4.705**	47.388	24.120	564.242	5.227	**30.160**
	Average queue	3.494	23.428	3.374	23.590	3.498	22.440	3.789	36.741	2.924	20.070	**1.568**	**15.796**	8.040	188.081	–	–
	Queue 1	0.146	10.926	0.189	11.022	0.129	11.437	0.646	28.921	0.140	10.753	0.341	10.019	11.106	65.160	–	–
	Queue 2	7.196	34.888	7.040	40.244	7.152	35.200	7.348	50.409	6.383	29.041	3.732	23.163	9.029	294.099	–	–
	Queue 3	3.141	24.470	2.894	19.504	3.214	20.684	3.373	30.894	2.248	20.417	0.632	14.206	3.985	204.984	–	–

**Table 3:** Performance comparison of different methods for the performance prediction of the GPS under higher burst traffic.

Utility of the Server	Methods	Decision_CART		Boosting_Xgboost		MLP		MLP1_Norm		GCN		DLPE		EBA_Multi		MPPA	
	Metrics	RMSE	MAPE	RMSE	MAPE	RMSE	MAPE	RMSE	MAPE	RMSE	MAPE	RMSE	MAPE	RMSE	MAPE	RMSE	MAPE
Ut = (0.7, 0.8)	GPS queue	0.794	33.342	0.262	24.557	0.325	103.707	0.776	142.814	0.712	55.628	**0.277**	**23.824**	8.037	510.786	0.532	82.498
	Average queue	0.265	11.114	0.087	8.186	0.108	34.569	0.259	47.605	0.237	18.543	**0.092**	**7.941**	2.679	170.262	–	–
	Queue 1	0.253	10.729	0.067	7.142	0.107	31.132	0.398	78.09	0.137	13.304	0.047	4.706	3.125	144.057	–	–
	Queue 2	0.242	10.773	0.095	8.674	0.109	35.773	0.221	40.79	0.321	22.846	0.111	6.838	2.750	188.550	–	–
	Queue 3	0.299	11.840	0.100	8.741	0.109	36.802	0.157	23.934	0.254	19.478	0.119	12.28	2.162	178.180	–	–
Ut = (0.8, 0.9)	GPS queue	1.124	43.674	0.714	25.873	0.821	59.717	1.151	108.249	**0.675**	32.012	0.807	**19.473**	32.700	145.361	1.111	29.788
	Average queue	0.375	14.558	0.238	8.624	0.274	19.906	0.384	36.083	**0.225**	10.671	0.269	**6.491**	10.900	48.454	–	–
	Queue 1	0.270	13.924	0.146	7.438	0.167	12.02	0.345	40.86	0.136	9.263	0.102	4.792	12.743	35.707	–	–
	Queue 2	0.532	14.820	0.332	9.279	0.414	31.501	0.456	39.012	0.291	11.396	0.298	5.643	8.891	55.380	–	–
	Queue 3	0.322	14.931	0.236	9.156	0.24	16.196	0.35	28.377	0.248	11.353	0.407	9.038	11.066	54.274	–	–
Ut = (0.9, 0.95)	GPS queue	**0.338**	36.473	0.427	**30.165**	0.474	54.739	0.898	103.795	1.046	55.446	1.730	63.018	3.148	215.842	0.424	42.577
	Average queue	**0.113**	12.158	0.142	**10.055**	0.158	18.246	0.299	34.598	0.349	18.482	0.577	21.006	1.049	71.947	–	–
	Queue 1	0.121	10.266	0.059	6.094	0.193	24.735	0.298	37.085	0.109	11.979	0.040	4.259	1.899	71.371	–	–
	Queue 2	0.096	12.845	0.273	13.255	0.231	22.934	0.385	38.707	0.867	33.801	1.658	54.425	0.793	83.299	–	–
	Queue 3	0.121	13.361	0.095	10.816	0.050	7.070	0.215	28.003	0.070	9.666	0.032	4.334	0.456	61.171	–	–

**Table 4:** Performance comparison of different methods for the performance prediction of the GPS under hybrid burst traffic.

Utility of the Server	Methods	Decision_CART		Boosting_Xgboost		MLP		MLP1_Norm		GCN		DLPE	
	Metrics	RMSE	MAPE	RMSE	MAPE	RMSE	MAPE	RMSE	MAPE	RMSE	MAPE	RMSE	MAPE
Ut = (0.7, 0.8)	GPS queue	1.817	35.324	1.057	23.298	1.269	68.845	1.268	59.329	1.086	58.139	**0.925**	**23.276**
	Average queue	0.606	11.775	0.352	7.766	0.423	22.948	0.423	19.776	0.362	19.380	**0.308**	**7.759**
	Queue 1	0.195	9.806	0.090	6.658	0.180	26.775	0.152	18.227	0.100	9.740	0.091	8.230
	Queue 2	0.667	12.992	0.342	8.264	0.389	13.661	0.405	20.556	0.352	23.649	0.302	6.461
	Queue 3	0.956	12.526	0.625	8.376	0.700	28.409	0.711	20.546	0.634	24.750	0.532	8.585
Ut = (0.8, 0.9)	GPS queue	2.138	50.139	1.398	**29.310**	1.603	79.850	1.733	87.859	1.379	43.704	**1.035**	34.939
	Average queue	0.713	16.713	0.466	**9.770**	0.534	26.617	0.578	29.286	0.460	14.568	**0.269**	11.646
	Queue 1	0.365	14.489	0.320	8.602	0.407	31.176	0.434	25.240	0.341	10.697	0.102	4.792
	Queue 2	0.727	15.152	0.715	10.318	0.781	17.291	0.831	32.649	0.666	14.945	0.298	5.643
	Queue 3	1.046	20.498	0.363	10.39	0.415	31.383	0.468	29.970	0.372	18.062	0.407	9.038
Ut = (0.9, 0.95)	GPS queue	2.760	72.729	2.625	78.986	2.440	86.612	3.374	121.628	2.351	52.921	**2.003**	**47.945**
	Average queue	0.920	24.243	0.875	26.329	0.813	28.871	1.125	40.543	0.784	17.640	**0.668**	**15.982**
	Queue 1	0.739	23.660	0.723	25.550	0.611	30.666	1.006	39.771	0.647	18.306	0.300	11.156
	Queue 2	1.011	24.749	0.909	27.013	0.970	29.437	1.203	42.688	0.798	16.945	0.934	20.172
	Queue 3	1.010	24.321	0.993	26.423	0.859	26.509	1.165	39.169	0.906	17.670	0.769	16.617

**Table 5:** Performance comparison of different methods for the performance prediction of the GPS under non-burst traffic.

Utility of the Server	Methods	Decision_CART		Boosting_Xgboost		MLP		MLP1_Norm		GCN	
	Metrics	RMSE	MAPE	RMSE	MAPE	RMSE	MAPE	RMSE	MAPE	RMSE	MAPE
Ut = (0.7, 0.8)	GPS queue	2.724	69.864	**1.749**	**62.785**	1.931	137.716	2.189	242.778	4.409	190.526
	Average queue	0.908	23.288	**0.583**	**20.928**	0.644	45.905	0.730	80.926	1.470	63.509
	Queue 1	0.157	14.380	0.181	18.405	0.288	35.753	0.382	68.040	1.681	65.080
	Queue 2	1.978	32.060	1.219	21.229	1.342	55.426	1.360	82.488	1.326	59.362
	Queue 3	0.589	23.424	0.349	23.151	0.301	46.537	0.447	92.250	1.402	66.084
Ut = (0.8, 0.9)	GPS queue	3.064	**54.818**	**2.307**	56.655	3.058	188.254	3.273	134.738	3.052	161.008
	Average queue	1.021	**18.273**	**0.769**	18.885	1.019	62.751	1.091	44.913	1.017	53.669
	Queue 1	1.077	18.029	0.893	18.060	1.098	57.326	1.184	42.322	1.165	56.523
	Queue 2	0.953	18.392	0.699	19.299	0.961	63.847	1.031	46.773	0.916	50.380
	Queue 3	1.034	18.397	0.715	19.296	0.999	67.081	1.058	45.643	0.971	54.105
Ut = (0.9, 0.95)	GPS queue	4.308	69.125	**3.906**	**58.554**	4.395	186.627	6.079	163.139	4.409	190.526
	Average queue	1.436	23.042	**1.302**	**19.518**	1.465	62.209	2.026	54.380	1.470	63.509
	Queue 1	1.441	22.375	1.463	18.945	1.570	56.631	2.204	54.974	1.681	65.080
	Queue 2	1.557	23.746	1.249	20.036	1.442	60.863	1.962	53.989	1.326	59.362
	Queue 3	1.310	23.004	1.194	19.573	1.383	69.133	1.913	54.176	1.402	66.084

**Table 6:** Performance comparison of different methods for the performance prediction of the GPS under heterogeneous traffic.

Utility of the Server	Methods	Decision_CART		Boosting_Xgboost		MLP		MLP1_Norm		GCN	
	Metrics	RMSE	MAPE	RMSE	MAPE	RMSE	MAPE	RMSE	MAPE	RMSE	MAPE
Ut = (0.7, 0.8)	GPS queue	**0.193**	**17.440**	0.765	32.583	1.097	189.256	0.736	154.571	0.929	192.627
	Average queue	**0.064**	**5.813**	0.255	10.861	0.366	63.085	0.245	51.524	0.310	64.209
	Queue 1	0.099	8.386	0.580	16.114	0.301	40.193	0.415	78.855	0.170	23.036
	Queue 2	0.033	4.417	0.057	7.876	0.597	97.846	0.233	60.555	0.640	144.916
	Queue 3	0.061	4.637	0.128	8.593	0.199	51.217	0.088	15.161	0.119	24.675
Ut = (0.8, 0.9)	GPS queue	0.708	38.107	**0.529**	**23.798**	1.516	154.923	0.933	112.942	0.988	135.806
	Average queue	0.236	12.702	**0.176**	**7.933**	0.505	51.641	0.311	37.647	0.329	45.269
	Queue 1	0.420	19.585	0.282	10.914	0.443	33.275	0.524	42.713	0.326	17.391
	Queue 2	0.216	9.275	0.195	6.151	0.854	82.241	0.325	56.362	0.526	97.965
	Queue 3	0.072	9.247	0.052	6.733	0.219	39.407	0.084	13.867	0.136	20.450
Ut = (0.9, 0.95)	GPS queue	**1.248**	**59.123**	1.295	60.963	2.534	197.001	1.989	145.675	1.698	130.040
	Average queue	**0.416**	**19.708**	0.432	20.321	0.845	65.667	0.663	48.558	0.566	43.347
	Queue 1	1.024	32.465	1.079	36.833	1.122	43.686	1.499	60.428	1.098	36.256
	Queue 2	0.115	12.780	0.101	11.507	1.103	102.901	0.353	70.648	0.387	71.791
	Queue 3	0.109	13.878	0.115	12.623	0.309	50.414	0.137	14.599	0.213	21.993

## Data Availability

Data are contained within the article.
